# Flexible loop and helix 2 domains of TCTP are the functional domains of dimerized TCTP

**DOI:** 10.1038/s41598-019-57064-9

**Published:** 2020-01-13

**Authors:** Heewon Lee, Mi-Sun Kim, Ji-Sun Lee, Hyunsoo Cho, Jimin Park, Dong Hae Shin, Kyunglim Lee

**Affiliations:** Graduate School of Pharmaceutical Sciences, College of Pharmacy, Ewha Womans University, Seoul, 03760 Korea

**Keywords:** Proteins, Structure determination

## Abstract

Translationally controlled tumor protein (TCTP), also called histamine releasing factor, is an evolutionarily conserved multifunctional protein in eukaryotes. We previously reported that extracellular TCTP acquires its cytokine-like function following dimerization. This study aims to identify the functional domain involved in the cytokine-like function of dimerized TCTP (dTCTP). We performed X-ray crystallographic studies and a deletion mutant of dTCTP which lacks the flexible loop domain. Synthetic peptides corresponding to TCTP domains and antibodies developed against them were examined for the anti-allergic effect. In an OVA-induced airway inflammation mouse model, inhibitory effect of synthetic peptides was evaluated. dTCTP was mediated by dimers between Cys172s of TCTP monomers. Synthetic peptides corresponding to the flexible loop and helix 2 domain of TCTP, and antibodies against them inhibited dTCTP-induced IL-8 release. In particular, the TCTP mutant lacking the flexible loop domain decreased the inflammatory cytokine activity of dTCTP. We conclude that the flexible loop and helix 2 domain of TCTP are the functional domains of dTCTP. They may have the potential to be therapeutic targets in the suppression of allergic reactions induced by dTCTP.

## Introduction

Histamine-releasing factor (HRF) was identified in the sera of allergic patients and it showed histamine releasing activity in human basophils, which in turn provoked allergic reactions^1,2^. Molecular identification studies revealed that histamine-releasing factor is identical to translationally controlled tumor protein (TCTP)^3^, a multifunctional protein that has been highly conserved in eukaryotes. In a previous study, we reported for the first time that dimerization allows TCTP to acquire cytokine-like activity^4^. TCTP exhibits several intracellular functions in development^5,6^, cell cycle regulation^7,8^, cellular homeostasis^9^, neurotransmitter release^10^ and autophagy^11^ and also in various diseases including cancer, malaria^12^, and Alzheimer’s diseases^13^.

Structural studies have revealed that TCTP possesses an intrinsically disordered structure consisting of three preserved motifs: a β-stranded sheet similar to that of Mss4/Dss4, a basic patch composed of H2-H3 helices^14^, and a flexible loop (FL) which comprises TCTP signature 1. The evolutionarily conserved motifs of TCTP indicate the importance of their functions. The fold of the β-stranded domain classifies TCTP as a member of Mss4-like superfamily; it plays a key role in eliciting TCTP’s GDP-GTP exchange activity for Rheb^15^. Its helical domain was identified as tubulin^16^ and calcium^17^ binding site and is reported to be involved in the inhibition of Bax dimerization due to its similarity with H5-H6 helices of Bax^18^. In a study of the possible function of the FL of TCTP, Yarm^8^ reported that a polo-like kinase reduces microtubule stabilizing activity of TCTP by phosphorylating Ser 46 and Ser 64 residues in the loop region of TCTP. However, the overall functions of the FL domain remain unknown.

Suggestions for NMR and crystal structures of TCTP have been deposited in the Protein Data Bank. These include different structures of monomeric TCTPs from several species and just one of human dimeric TCTP (dTCTP). They all share a high topological similarity. In both forms, a long middle loop located outside of the core domain of TCTP shows high flexibility. dTCTP shows a unique dimeric interface which seems to be further stabilized by the disulfide bond mediated by Cys^172^ ^19^. Structural and functional information of the long loop is currently still very limited.

In this study, we examined which domains of dTCTP are important for its function by X-ray crystallographic studies. We also studied the function of the FL of TCTP using a deletion mutant. Based on the structures of human dTCTP and this mutant, we predicted the mechanism of action of each functional domain.

## Materials and Methods

### Preparation of recombinant proteins and synthetic peptides

The sequences coding human TCTP, **∆-**TCTP (without residues Arg^38^-Val^66^), rat recombinant TCTP (RrTCTP), 11–172 residues-deleted RrTCTP (Del-N11dTCTP), and FL-deleted Del-N11dTCTP (∆-del-N11dTCTP) were amplified for cloning into bacterial expression vector. Detailed information for gene cloning is described in Supporting information (SI, Table S1). Clones were transformed into *E. coli* BL21 (DE3) for protein expression. Overexpressed protein was purified using a HisTrap column on an ÄKTA-explorer system (GE Healthcare), followed by ion-exchange chromatography using a Hi-Trap Q column (GE Healthcare). Peptides were synthesized by Fmoc solid-phase method by AbClon or Peptron Inc. N-terminal free amine groups were acetylated, and the C-terminal free carboxyl groups were amidated to improve the stability of the peptides. Sequences of each peptide are displayed in SI (Table S2).

### Productioin of full length human TCTP and FL domain deleted mutant TCTP dimers

For producing homogenous monomeric form, 10 μg of each protein in 10 μl was treated with 0.1–10 mM 1,4-dithiothreitol (DTT) and incubated at room temperature for 30 minutes or 24 hours. For producing homogenous dimeric form, 10 μg of each protein in 10 μl was treated with 1–100 mM of tertiary-butyl hydroxide (t-BH) or H_2_O_2_ and incubated at room temperature for 30 minutes or 24 hours. Protein samples were analyzed in 15% non-reducing or reducing gel. After SDS-PAGE, gels were subjected to either Coomassie blue staining or immunoblotting using antibodies against flexible loop and helix 2 domain.

### Cell culture

BEAS-2B, a human bronchial epithelial cell line, was purchased from the American Type Culture Collection (ATCC, CRL-9609). Cells were maintained in bronchial epithelial cell growth medium (BEGM, Lonza) at 37 °C and 5% CO_2_.

### Animal model of OVA-induced airway inflammation

All animal studies were approved by Ewha Womans University’s Institutional Animal Care and Use Committee (IACUC, approval ID: 16-023). All methods and experimental procedures were conducted according to the guidelines of the Ewha Womans University’s IACUC. The animals were housed under pathogen-free conditions with a 12-h light/12-h dark cycle, and were fed with standard diet and water *ad libitum*. 5 weeks old BALB/c female mice (Orientbio) were sensitized with OVA by i.p. injection of 1.3 mg of aluminum hydroxide (Sigma) and 100 µg of ovalbumin (Sigma) twice a week, followed by challenge with intranasal instillation of OVA on day 14, 16, and 18. Ten minutes before challenge, PBS, dexamethasone, and synthetic peptides were intraperitoneally injected. On days 15, 17, and 19, i.p administration was performed without antigen administration. On day 19, the animals were sacrificed 2 hours after the i.p injection, followed by BALF and lung tissue preparation.

### Crystal structure analysis

Details for structure determination are described in SI (Table S3).

### Statistical analysis

Data were analyzed using GraphPad Prisms 5 software and expressed as mean ± standard error of mean (SEM). Statistical significance was determined using Student’s two-tailed unpaired t-test for comparison between two groups. For three or more groups, one-way ANOVA analysis was performed.

## Results

### Production of full length TCTP (f-TCTP) and FL domain deleted mutant of TCTP (∆-TCTP) in monomeric and dimeric forms

**Figure 1:**
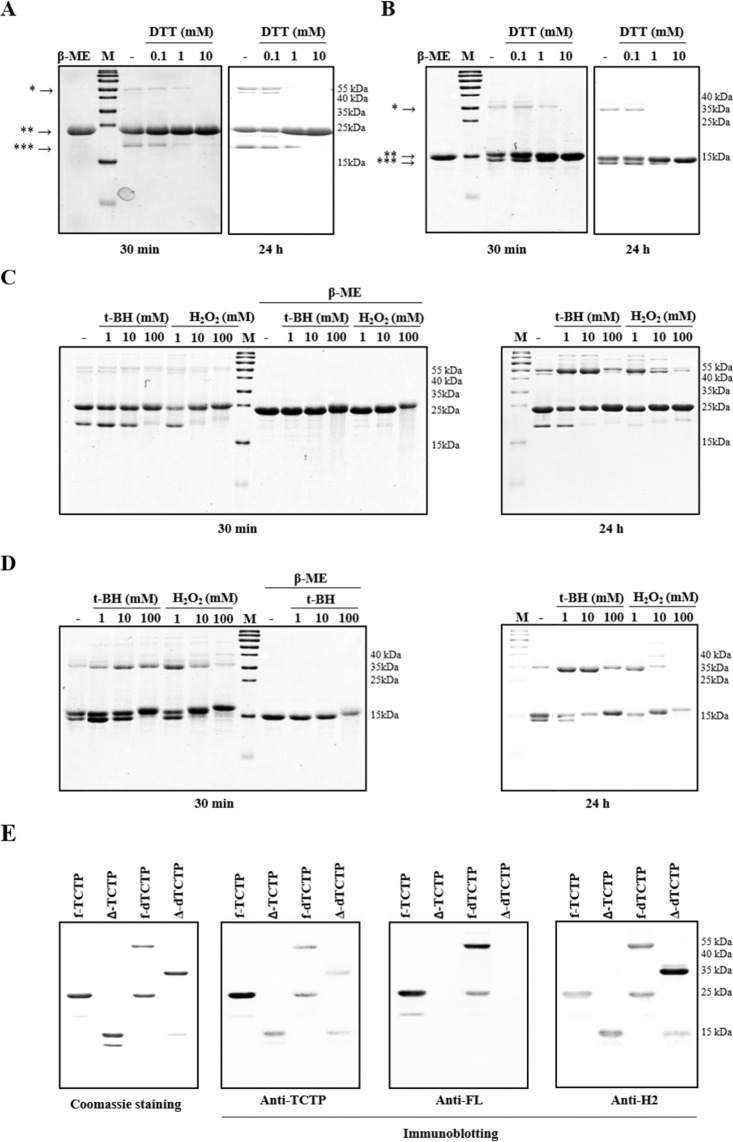
Full length human TCTP and FL domain deleted mutant TCTP form dimers. (**A**,**B**) Each recombinant protein was treated with DTT at room temperature for 30 minutes or 24 hours to obtain purified f-TCTP (**A**) and Δ-TCTP (**B**) as homogeneous monomers. *homodimer, **monomer, ***monomer with intramolecular disulfide bond. (**C**,**D**) The conditions for maximizing the ratio of the dimer were investigated by treating 10 μg of purified f-TCTP (**B**) and Δ-TCTP (**D**) with t-BH or H_2_O_2_ for 30 minutes or 24 hours at room temperature. (**E**) Monomer and dimer forms of f-TCTP and Δ-TCTP. Dimer formed by treatment of 10 mM t-BH for 24 hours was analyzed on 15% SDS-PAGE gel and subjected to Coomassie staining or immunoblotting using TCTP, FL (Anti-FL) and H2 (Anti-H2) antibody. M: molecular weight marker.

Several studies have investigated the role of the intrinsically disordered proteins (IDPs) in cellular interactions under the physiological conditions^20–23^. These structurally diverse proteins can produce biological responses by binding to or providing structured hub proteins with potential partner proteins^24^. Considering the intrinsically disordered structure of TCTP^15^, this structural characteristic may be important to elicit the biological function of TCTP. In order to understand the role of FL domain in dimeric TCTP, we constructed full length human TCTP (f-TCTP) and the FL domain deleted mutant TCTP (∆-TCTP) expression vectors and produced recombinant f-TCTP and Δ-TCTP proteins. Purified proteins were analyzed by SDS-polyacrylamide gels both in reducing and non-reducing conditions. We found that the molecular weights of most of the expressed proteins corresponded to the monomers (25 kDa for f-TCTP and 17 kDa for Δ-TCTP), but two weak bands were also observed (Fig. 1A,B). These bands were confirmed to be a homodimer and monomer produced by intermolecular or intramolecular disulfide bonding between Cys^28^ and Cys^172^ which disappeared upon addition of reducing agents, beta-mercaptoethanol (β-ME) and DTT. When both f-TCTP and ∆-TCTP were treated with 1 mM DTT for 24 hours, homodimers were reduced to monomers, but monomers with intramolecular disulfide bonds remained. However, at 10 mM, DTT completely reduced both proteins regardless of incubation time.

We then analyzed dimer formation by each protein using the oxidizing agents, t-BH and H_2_O_2_ (Fig. 1C,D). At a given concentration, longer reaction times led to more dimer formation. When the reaction time was 30 minutes, f-TCTP remained mostly monomeric regardless of the type and concentration of the oxidizing agent, but when the reaction time was extended to 24 hours, dimers were formed even at the lowest concentration (Fig. 1C). ∆-TCTP was more susceptible to dimerization than f-TCTP under the same conditions (Fig. 1D). We also confirmed that dimerized f-TCTP and ∆-TCTP were reduced to monomers by β-ME. Unexpectedly, proteins treated with 100 mM t-BH or 10 mM H_2_O_2_ or higher reduced dimer yield. In addition, the molecular weight of monomers and dimers formed under these conditions was found to be slightly increased, which might be due to the presence of other oxidation sites in addition to the sulfides of Cys residues. We further analyzed the t-BH-induced formation of monomers or dimers by coomassie blue staining and immunoblotting (Fig. 1E), and confirmed the deletion of the FL in Δ-TCTP and presence of helix 2 (H2) domain in all proteins.

### The lack of the FL domain in dTCTP inhibits its ability to release cytokines

**Figure 2:**
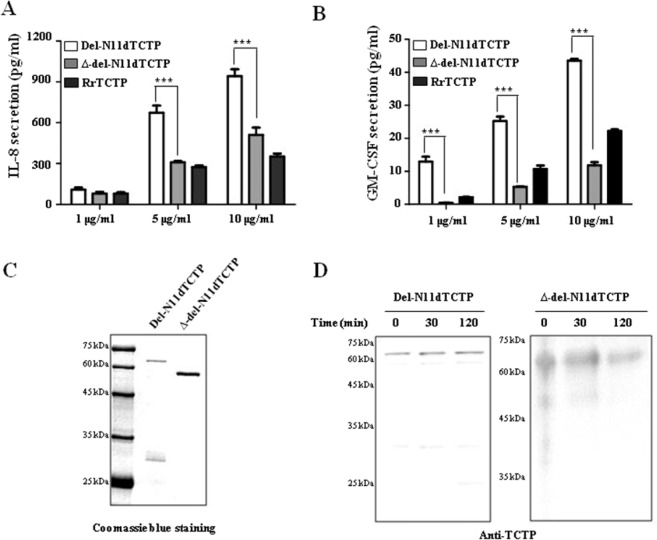
Lack of the FL in dimeric TCTP inhibits release of cytokine in BEAS-2B cells. (**A**,**B**) BEAS-2B cells were treated with indicated dose of RrTCTP, del-N11dTCTP or ∆-del-N11dTCTP for 24 h. The IL-8 (**A**) or GM-CSF (**B**) in the supernatant was analyzed by ELISA. (**C**) Each purified protein was analyzed on SDS-PAGE gel in non-reducing condition. First lane in each membrane is molecular weight size marker. (**D**) Each purified protein was incubated in supernatant from cultured BEAS-2B cells for 30 and 120 min at 37 °C, followed by non-reducing SDS-PAGE analysis for confirmation of dimerization. Values represent mean ± SEM, ***p < 0.001.

Previous studies have shown that dimeric TCTP formed by N-terminal deletion (del-N11dTCTP) secreted the inflammatory cytokines, IL-8 and GM-CSF^4^ and also endows it with stronger effects when compared to monomeric RrTCTP (Fig. 2A,B). To understand the role of FL domain in the induction of inflammatory response by dTCTP, we compared IL-8 and GM-CSF secretion capacities of del-N11dTCTP and FL deleted del-N11dTCTP dimer (∆-del-N11dTCTP) in BEAS-2B cells. Cells treated with ∆-del-N11dTCTP showed decreased secretion of IL-8 and GM-CSF when compared to cells treated with del-N11dTCTP (Fig. 2A,B), suggesting that FL is involved in the inflammatory response of del-N11dTCTP. Dimeric status of del-N11DTCTP and ∆-del-N11dTCTP protein was confirmed by coomassie blue staining. Both proteins showed shifted mobility corresponding to dimers in non-reducing condition (Fig. 2C). We further confirmed that the dimeric status of del-N11dTCTP and ∆-del-N11dTCTP were maintained in supernatants from cultured BEAS-2B cells by western blotting using anti-TCTP antibody (Fig. 2D). These findings demonstrate that FL domain enables dTCTP to provoke inflammatory cytokine-releasing activity.

### Antibodies against synthetic peptides corresponding to the FL and H2 domains, inhibit the cytokine-like activity of dTCTP *in vitro*

**Figure 3:**
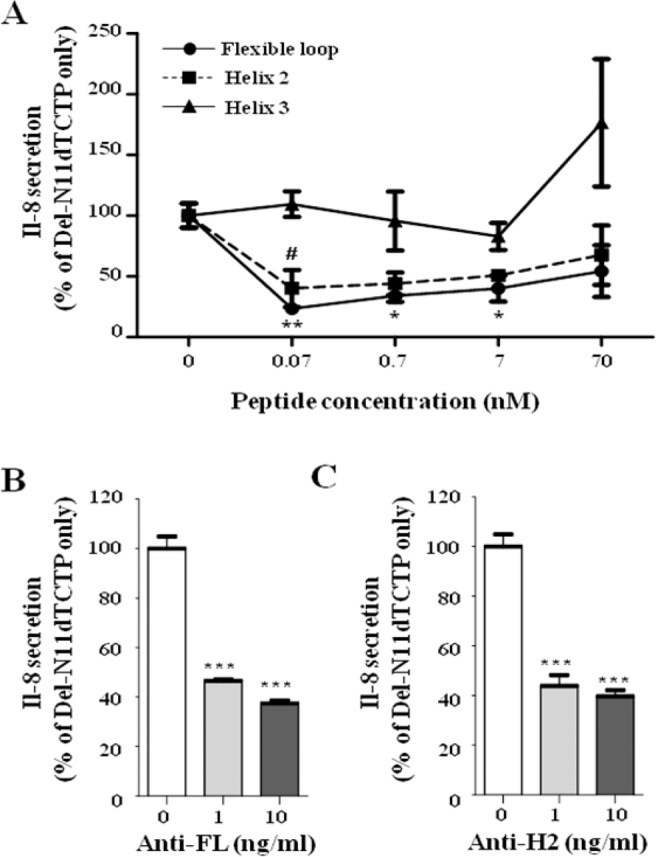
Both FL and H2 peptides and antibodies against them inhibit del-N11dTCTP-induced IL-8 release. (**A**) Synthetic peptides of FL, H2, or H3 domain were pre-treated to the BEAS-2B cells at 70 pM to 70 nM. After 30 min, del-N11dTCTP (70 nM) was treated to the cells and incubated for 16–20 hours. The IL-8 in the supernatant was quantified by ELISA. Values represent mean ± SEM, n = 3. *p < 0.05, **p < 0.01; vs 0 nM in FL treated groups, ^#^p < 0.05; vs 0 nM in H2 treated groups. (**B**,**C**) Del-N11dTCTP (70 nM) and indicated doses of anti-FL antibody (**B**) or anti-H2 antibody (**C**) were incubated for 30 min. The mixture was then treated to the BEAS-2B cells for 16–20 hours. The IL-8 in the supernatant was quantified by ELISA. Values represent mean ± SEM, n = 3. ***p < 0.001; 0 vs 10 ng/ml.

dTBP2, a 7mer peptide that inhibits the function of dimerized TCTP was found to bind to the H2 domain corresponding to the 89–110 sequence of murine TCTP in domain maping experiments^25^. Therefore, we deduced that the FL and H2 domains could be important for the function of dimerized TCTP. To verify this hypothesis, peptides corresponding to the FL, H2, and helix 3 (H3) domains and polyclonal antibodies against them were tested for their inhibitory effect on del-N11dTCTP-induced cytokine release. IL-8 release by del-N11dTCTP was significantly reduced in BEAS-2B cells pretreated with 0.07, 0.7, and 7 nM FL peptides and 0.07 nM H2 peptides (Fig. 3A). At 0.07 nM concentration, both peptides significantly reduced IL-8 secretion induced by Del-N11dTCTP. As a control, we used a peptide derived from the H3 domain of the TCTP and it did not affect the IL-8 secretion. Furthermore, 1 and 10 ng/ml of anti-FL antibody reduced IL-8 secretion by 53.5% and 62.6%, respectively (Fig. 3B), and the same concentrations of anti-H2 antibody reduced the level of IL-8 to 43.8% and 39.6% (Fig. 3C), respectively.

### Synthetic peptides of FL and H2 domains exhibit anti-allergic effects in OVA-induced airway inflammation mouse model

**Figure 4:**
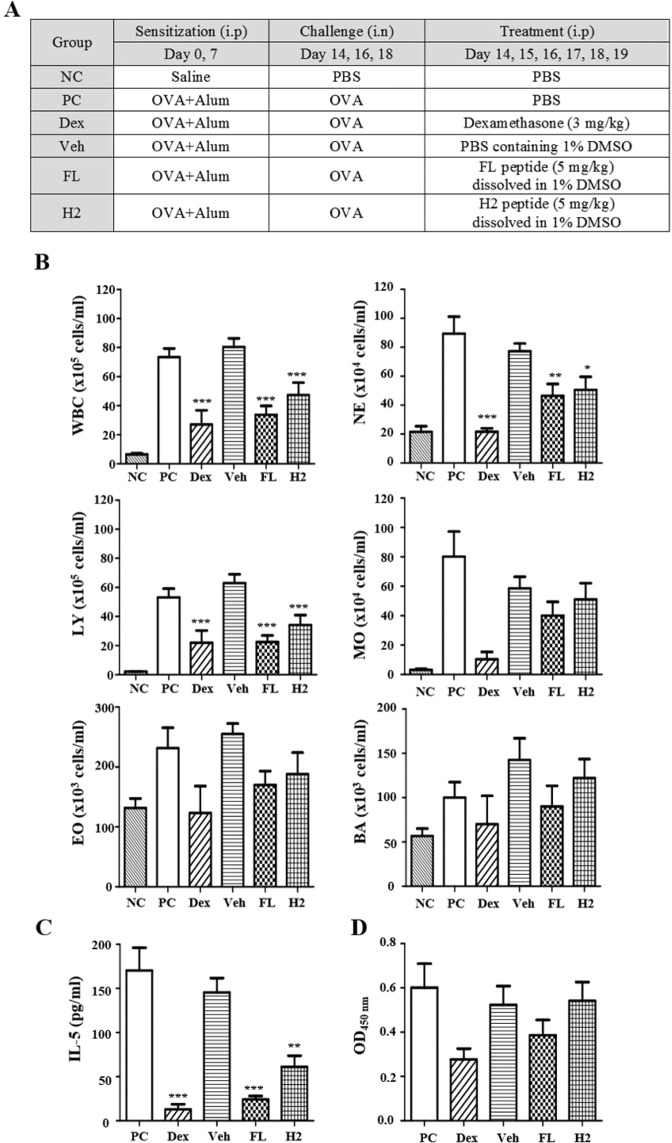
Anti-inflammatory effect of FL and H2 peptides in an OVA-induced airway inflammation model. (**A**) Description of OVA-induced airway inflammation model. Female Balb/c mice were sensitized with OVA allergen or vehicle. Two weeks after the sensitization, mice were challenged with allergen or vehicle and received an i.p. injection of PBS, dexamethasone (3 mg/kg), vehicle, FL or H2 peptide (5 mg/kg) on indicated days. (**B**) Inflammatory cells infiltrating into the bronchus were measured in BALF using HEMAVET 950FS. (**C**) IL-5 level in BALF was measured using ELISA. (**D**) OVA-specific IgE in serum was measured using ELISA. NC: negative control (n = 6), PC: positive control (n = 6), Dex: dexamethasone (n = 3), Veh: vehicle (1% DMSO, n = 4), FL: FL peptide (n = 5), H2: H2 peptide (n = 5), WBC: white blood cells, NE: neutrophils, LY: lymphocytes, MO: monocytes, EO: eosinophils, BA: basophils. Values represent mean ± SEM, *p < 0.05, **p < 0.01 ***p < 0.001; compared to PC.

Airway inflammation is characterized by the infiltration of inflammatory cells such as neutrophils, basophils and eosinophils, into the bronchi. These cells secrete cytokines such as IL-4, IL-5 and IL-13, promote differentiation of B cells, and further activate the immune system^26–28^. It has been reported that OVA challenge promotes extracellular secretion of TCTP, and that dTCTP induced by N-terminal truncation can induce airway inflammation^4,29^. Therefore, we tested the effect of FL or H2 peptides in a mouse model of OVA-induced airway inflammation (Fig. 4A). We sensitized balb/c mice with OVA + Alum and administered each peptide before challenge. Dexamethasone was used as a representative anti-inflammatory reagent in this experiment. As a criterion for inflammatory response, we analyzed inflammatory cell numbers, an IL-5 in bronchoalveolar lavage fluid (BALF). Each peptide significantly reduced infiltration of inflammatory cells compared to the positive control group (Fig. 4B). Especially, numbers of white blood cells, neutrohils, and lymphocytes were significantly decreased by each peptide treatment. Moreover, IL-5 was significantly reduced by FL and H2 peptide treatments, indicating that these peptides were exerting a blocking effect on inflammatory response (Fig. 4C). Even though there is no statistically significant difference between groups, the serum concentration of OVA antigen-specific IgE also can be stated to decrease in the FL peptide-treated group (Fig. 4D).

However, this experiment has the limitation that the study included no control for each peptide. The net charges of FL and H2 peptides are −6 and +4, respectively, suggesting possibility that the anti-inflammatory effects observed here may be due not only to competition with TCTP, but also due to non-specific interaction with the opposite electrical charge. Indeed, a study reported that a peptide with a +4 charge did not affect OVA-induced airway inflammation^30^. These findings suggest that the peptides in the FL and H2 domain may be important therapeutic targets for airway inflammation, but admittedly the data in Fig. 4 should be interpreted with caution.

**Figure 5:**
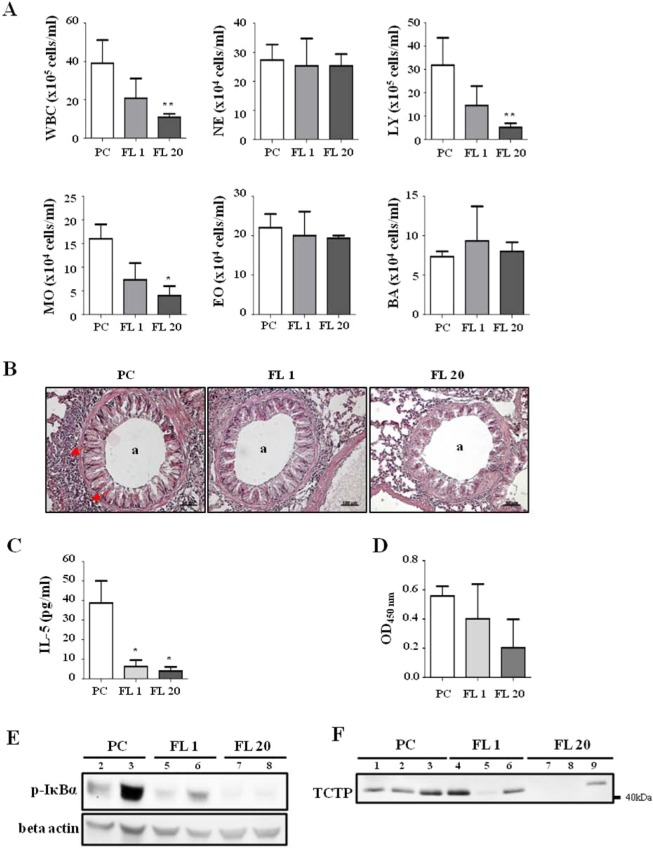
Dose-dependence of anti-inflammatory effect of FL peptide. (**A**) Inflammatory cells that infiltrated to the bronchus were measured in BALF using HEMAVET 950FS. (**B**) H&E staining of lung tissues was performed to visualize cell infiltration. The *a* indicates the airway, and red arrows indicate inflammatory infiltrates. (**C**) IL-5 level in BALF was measured using ELISA. (**D**) OVA-specific IgE in serum was measured using ELISA. (**E**) Lung tissue was homogenized and immunoblotted with phospho IκBα and beta actin antibodies. (**F**) BALF was concentrated and immunoblotted for TCTP. Each lane represents biological replicate indicated by the number. PC: positive control (n = 3), FL 1: FL 1 mg/kg (n = 3), FL 20: FL 20 mg/kg (n = 3), WBC: white blood cells, NE: neutrophils, LY: lymphocytes, MO: monocytes, EO: eosinophils, BA: basophils. Values represent mean ± SEM, *p < 0.05, **p < 0.01; compared to PC.

Then we examined the dose-dependence of FL peptide in the mouse model. Figure 5A shows a decrease in inflammatory cell infiltration according to FL peptide treatment. We examined the histological changes of lung tissues in each group after H&E staining (Fig. 5B). In the positive control group, leukocytes were found infiltrated into and around the bronchiole and mucous membrane, resulting in hyperplasia in epithelial cells. Though the changes in 1 mg/kg FL peptide treated group were not significant compared to positive control group, there was an obvious reduction in infiltrated leukocytes in dose of 20 mg/kg. The IL-5 levels in the BALF of the mice treated with 1 mg/kg or 20 mg/kg of FL peptide decreased to 16% and 10.2% of control group, respectively (Fig. 5C). Although there was no statistical significance, serum OVA-specific IgE also tended to decrease in a dose dependent fashion (Fig. 5D). Because NF-κB pathway has been shown to be induced by del-N11dTCTP^31^, we examined IκBα phoshorylation in the lung tissue (Fig. 5E). We found that IκBα phosphorylation decreased following FL peptide treatment. When analyzing TCTP in BALF, two strong bands were detected at above 40 kDa which correlates with dimeric TCTP (Fig. 5F), and above 70 kDa (data not shown). These physiological forms of extracellular TCTP tended to decrease following FL peptide treatment. Dose dependency is not clearly established since each group consists of only three mice. However, TCTP was not detected in 1 of 3 FL1 and 2 of 3 FL20 groups. Extracellular TCTP was significantly produced in the BALF of the positive group and decreased by the administration of the FL peptide, which in turn corresponded with the decrease in leukocyte infiltration and IL-5 levels in the BALF and the decrease in IκBα phosphorylation in the lung tissue. From these results, the amount of extracellular TCTP observed can be assumed to reflect the degree of inflammation in the mouse experimental model. These results confirm the role of FL in controlling the inflammatory response.

### Crystral structures of f-dTCTP and ∆-dTCTP reveal that limited movement of FL is critical for stable dimerization and its function

We previously reported that del-N11dTCTP dimerizes through an intermolecular disulfide bond with cytokine releasing activity^3^. For the structural study, we attempted to crystallize several NH_2_-terminus truncated forms, but found that proteins were poorly overexpressed in *E. coli*. Consequently, our crystallogpraphic studies were done with f-TCTP and ∆-TCTP which were well overexpressed in *E. coli*.

**Figure 6:**
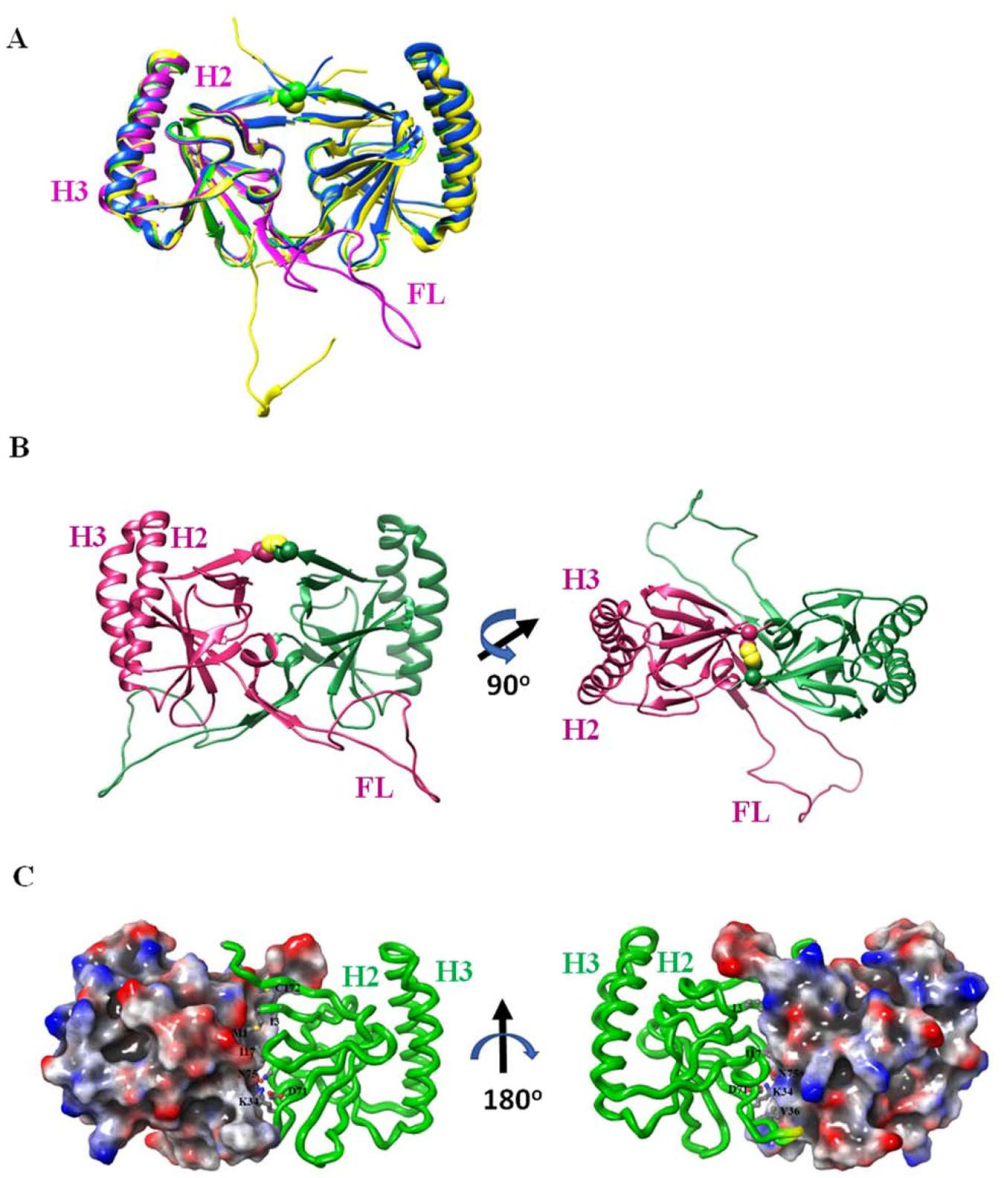
Crystal structures of f-dTCTP and ∆-dTCTP. (**A**) The comparison of 3D-structures of f-dTCTP (green) and Δ-dTCTP (blue) indicates that their overall architecture is conserved. They form a dimer mediated by Cys^172^ (yellow) which is depicted with a sphere model based on the crystal structure of f-dTCTP. The previously reported dimeric X-ray structure (5O9M, yellow) and a monomeric NMR structure (2HR9, pink) are also superimposed. (**B**) The modeled crystal structure of dimeric TCTP. Two subunits are colored in green and deep pink. The FL of TCTP determined by NMR is fused to the crystal structure of f-dTCTP where its FL is not determined. Among the NMR structures, a model avoiding collision when a dimeric form was constructed was selected. The modeled f-dTCTP is built by inserting the chosen FL between Ser37 and Ile67 of f-dTCTP. The right figure was drawn by rotating 90 degree of the left model. (**C**) The crystal structure of Δ-dTCTP. The crystal structure of Δ-dTCTP is drawn with an electrostatic potential surface (gradient from blue (positive) to red (negative)) of one subunit and a ribbon model (green) for the other. The residues on the dimeric interface are labeled and drawn with a ball-and-stick model. The secondary structure elements were also labeled with β for β-strands and H for α-helices. In the right figure, the yellow region of the ribbon model represents two glycine residues located on the loop between β5 and β6 (almost hidden in this view) genetically engineered to connect Ser^37^ and Ile^67^.

Until now, five human TCTP structures (PDB ID: 5O9L, 5O9M, 1YZ1, 2HR9 and 3EBM) determined by X-ray crystallogrphy or NMR have been reported. Interestingly, three of these suggested possible dimeric forms have been based on the packing in the different unit cells^32^. Among them, a dimeric form mediated by Cys172 has been suggested to be a biological dimer which accords with reported biochemical results (Fig. 6A). Our crystal strucutre of f-dTCTP obtained under oxidative condition also accords with this disulfied-bridge mediated dimeric form with RMSD values for 286 Cα atoms of ~0.85 Å.

Several structural properties of the dimeric forms are of particular interest (Fig. 6B). First, the interaction at the dimeric interface of dTCTP is not strong. The total accessible surface area buried at each binding site is ~ 640 Å^2^ per each subunit without a hydrophobic core or a salt- bridge network. Therefore, the dimeric forms of dTCTP may be more stable when the the disulfide bond is formed. Second, dTCTP takes an extended conformation and shows a butterfly like shape. As a result, two coiled-coil like helices (H2 and H3) are located far apart from each other, and two Fab binding sites (residues 1–19 on the N-terminal and residues 107–135 on H3)^33^ become surface exposed in each molecule. Third, the flexible loops are positioned in the opposite sides of their N- and C-terminals. Consequently, if a dimer formed, the movement of the FL is restricted at one side. In contrast, the movement of the FL of the monomeric form is expected to reach the region forming the dimeric interface and thus obstructs dimer formation. The NMR structures explicitly support this possibility^34^. Therefore, to sustain the dimeric state, the disulfide bond may be critical to overcome the pressure from the movement of the FL hampering dimer formation. Conversely, the limited flexibility of the FL at the confined space in the dimeric form may be necessary for its molecular function.

In order to investigate the influence of the FL over the overall structure of f-dTCTP, we determined the X-ray crystal structrue of Δ-dTCTP at 1.90 Å resolution (Fig. 6C). The overall architecture of Δ-TCTP structure comprises four β-sheets, designated β1– β4, and three main helices, designated H1–H3, all of which are found in f-TCTP. The first β-sheet is composed of three β-strands β2, β1, β11, the second with β3, β4, β10, the third with β7, β8, β9 and the last with β5, β6. Among them, the last one does not form an ideal β-sheet due to the influence from the deletion of the FL. The Δ-TCTP is superimposed on f-TCTP with RMSD values for 143 Cα atoms of ~0.4 Å. In the unit cell, two Δ-TCTPs also form a dimer connected by the disulfide-bridge between Cys^172^ residues and show the same dimeric archtecture found in the f-dTCTP. Therefore, Δ-dTCTP is also superimposed on f-dTCTP with RMSD values for 286 Cα atoms of ~0.8 Å. The uneven distribution of electrostatic potential caused by ten lysine and two arginine residues concentrated on H2 and H3 is evident as shown in Fig. 6C. In summary, the FL does not influence to the overall structure of dTCTP. Collectively, these data indicate that limitation of the flexibility of the FL by the disulfide bond is critical for molecular function of dTCTP even though this does not affect the overall structure of dTCTP.

## Discussion

This study found that the mutant TCTP lacking the FL domain (∆-del-N11dTCTP) exhibits impaired cytokine-like activity of del-N11dTCTP, even if it becomes a dimer. In addition, the synthetic peptide that competes with the FL domain of dTCTP for its putative receptor, and anti-FL antibody which can mask the loop domain of dTCTP, could effectively inhibit the dTCTP-induced cytokine release. The reported structure of dimeric fTCTP indicates that the shapes of the dimeric forms are like a butterfly in which the interface is formed with a weak interaction between edge of β-sheet. The crystal structure of the dimeric Δ-TCTP determined in this study shows no overall structural difference except for the presence of loop domain. Therefore, the function of the dTCTP appears to be largely due to the FL domain of the dimeric TCTP.

The FL domain of TCTP contains one of the conserved sequences of the TCTP protein family and has been reported to be highly mobile and disordered. The first identified backbone structure of the TCTP of *Schizosaccharomyces pombe*, p23^*fyp*^ ^35^, suggested that molecular interactions of this loop would play a crucial role in the function and regulation of this protein family. It is now well accepted that IDPs perform important biological functions without stable tertiary structures^36^. One of the important functions of IDPs is molecular recognition, and there is growing evidence that IDPs mediate ligand-receptor binding^37^. Examples of receptors involved in ligand-receptor interactions include glucocorticoid receptor^38^, growth hormone receptor^39^, and retinoid X receptor^40^. Multiple phosphorylations of IDP have been reported to cause protein-protein interactions by modulating folding of IDP^41,42^. These reports suggest that the phosphorylation of Ser^46^, Ser^64^ and Thr^65^ by plk-1 not only affects the microtubule-stabilizing activity of TCTP, but also induces conformational change in the loop region, thus providing the possibility of interacting with other targets.

Monomeric TCTP (mTCTP) also contains the FL domain, but it does not exhibit cytokine-like function probably due to the differences in the orientations of the loop domains in mTCTP and dTCTP. In mTCTP, the FL domain is thought to be disordered and free to move^14,35^. But when it forms a dimer, the free movement of the FL is restricted in a certain confined space. This structural difference provides an explanation for the previously reported higher affinity of dTBP2 for dimers than for monomers^25^. The FL of mTCTP can move freely and, to some extent, prevent dTBP2 from binding to the H2 region. However, for dTCTP, the loop region is directional and does not affect the binding of dTBP2.

Based on the fact that the dTBP2 peptide, interacting with the H2 site, showed anti-allergic effects in mouse models^25,43^, the H2 domain was assumed to be another functional domain. We examined the effect of a synthetic peptide corresponding the H2 domain and antibody against it on dTCTP-induced cytokine secretion. As expected, IL-8 secretion decreased when the H2 region of dTCTP was interrupted, suggesting that the H2 region plays an important role in the cytokine-like function of dTCTP. To more clear illustrate the function of the H2 region, construction of a deletion mutant lacking the H2 domain was considered. However, the H2 domain was found to be an important axis for the overall structure of dTCTP, forming a coiled-coil structure with the H3 region. Therefore, the mutant would not reflect H2 domain-specific functions, but rather overall mutation in TCTP structure.

## Conclusion

This research, for the first time, demonstrated the important role of the FL and H2 domains of TCTP in dTCTP-induced inflammation. In conclusion, we hereby propose that the FL and H2 domains are the functional domains of dTCTP and promising therapeutic targets for allergic diseases.

## Supplementary information

supplementary information.
