# Supplementary material for: Flexible loop and helix 2 domains of TCTP are the functional domains of dimerized TCTP
Source: Sci Rep. 2020 Jan 13;10:197. doi: 10.1038/s41598-019-57064-9 (PMC6957494; doi:10.1038/s41598-019-57064-9)
Supplement: Supplementary file 1 — supplementary information. [file 41598_2019_57064_MOESM1_ESM.docx]

**Flexible loop and helix 2 domains of TCTP are the functional domains of dimerized TCTP**

Heewon Lee^1,a^, Mi-Sun Kim^1,a^, Ji-Sun Lee^a^, Hyunsoo Cho^a^, Jimin Park^a^, Dong Hae Shin ^a,b^ and Kyunglim Lee ^a,b^

^a^ Graduate School of Pharmaceutical Sciences, College of Pharmacy, Ewha Womans University, Seoul 03760, Korea

^1^ These authors contributed equally to this work.

^b^ To whom correspondence should be addressed: Dr. Dong Hae Shin and Dr. Kyunglim Lee, Graduate School of Pharmaceutical Sciences, College of Pharmacy, Ewha Womans University, Seoul 03760, Korea Tel: 82-2-3277- 4502, 82-2-3277-3024

Email: [dhshin55@ewha.ac.kr](mailto:dhshin55@ewha.ac.kr), [klyoon@ewha.ac.kr](mailto:klyoon@ewha.ac.kr)

**SUPPORTING INFORMATION**

**Table S1. Primers and vectors for cloning**

| Construct | Primer | Vector | Tag protein |
| --- | --- | --- | --- |
| f-TCTP, Δ-TCTP | *forward*-GGAATTCCATATG(*NdeI*)ATTATCTACCGGGAC  *reverse*- CCGCTCGAG(*XhoI*)ACATTTTTCCATTTC TAA | pET22b (Novagen) | C-terminal His_6_ tag |
| Rat TCTP | *forward*-CG GGATCC(*BamHI*)ATGATCATCTACCGGGAC  *reverse*-CCCAAGCTT(HindIII)ACATTTTTCCATCTCTAA GCC | pRSET A  (Invitrogen) | N-terminal His_6_ tag |
| Del-N11dTCTP,  ∆-del-N11dTCTP | *forward*- CGGGATCC(*BamHI*)GACGAGCTGTCCTCCGACAT  *reverse*- CCCAAGCTT(*HindIII*)ACATTTTTCCATCTCTAA | pRSET A  (Invitrogen) | N-terminal His_6_ tag |

**Table S2. Peptide sequences**

| Peptide | Sequence |
| --- | --- |
| FL | SRTEGAIDDSLIGGNASAEGPEGEGTESTVVT |
| H2 | TKEAYKKYIKDYMKSLKGKLEEQKP |
| H3 | KPERVKPFMTGAAEQIKHILANFN |

**Structure determination**

The sequences coding human TCTP and ∆-TCTP for crystallizaton were cloned using the bacterial expression vector, pET22b (Novagen) prducing noncleavable C-terminal His_6_-tag. For ∆-TCTP, a FL (residues Arg^38^ ~ Val^66^) was deleted and two glyine resdidues connecting Ser37 and Ile67 was artificially included. Each clone was transformed into DH5α competent cells, screened by plasmid DNA analysis and transformed into *E. coli* BL21(DE3) for protein expression. Proteins were purified and subjected to ion-exchange chromatography and the finally purified proteins were treated with 1 mM tert-butylhydroxide before crystallization trials. Diffraction-quality crystals of f-TCTP were obtained using a reservoir solution (0.1 M MES pH 6.5, 0.2 M ammonium acetate, 25% PEG1500). Crystals grew to dimensions of 0.2 x 0.05 x 0.05 mm within a week at 296 K. Based on the Matthews coefficient V _M_ (Å^3^/Da) of 1.87, the asymmetric unit could contain four TCTP protomer with a solvent content of 34.09 %. The crystals of ∆-TCTP were obtained using a reservoir solution (0.1 M MES pH 6.5, 25% PEG300) and grew to dimensions of 0.03 x 0.05 x 0.05 mm within a week at 296 K. Based on the Matthews coefficient V _M_ (Å^3^/Da) of 2.86, the asymmetric unit could contain one ∆-TCTP protomer with a solvent content of 57.04 %. The details of the data-collection statistics are presented in Table S3

Molecular replacement (MR) was performed using PHENIX with the monomeric form of the X-ray crystal structure of human TCTP (PDB ID: 1YZ1) as a search model in both cases. Iterative density modification, model building and refinement were performed using the AutoBuild wizard of PHENIX. The model refinement together with addition of water molecules were performed using COOT and phenix refine. All the residues of f-dTCTP were visible except the FL. The model of f-∆TCTP was also well defined including two glycine residues genetically engineered. Both final models exhibited good stereochemical geometry. All the residues lie in the allowed region of the Ramachandran plot. The details of the refinement statistics are provided in Table S3. The atomic coordinates and structure factors of f-dTCTP and ∆-dTCTP have been deposited in the Protein Data Bank with the codes 6IZE and 6IZB, respectively.

## Table S3. Data collection and refinement statistics.

| Molecule (PDB ID) | f-dTCTP (6IZE) | Δ-dTCTP(6IZB) |
| --- | --- | --- |
| Wavelength (Å) | 0.97933 | 1.00000 |
| Resolution range* (Å) | 55.72 - 2.29 (2.37 - 2.29) | 46.37 - 1.90 (1.97 - 1.90) |
| Space group | P 4_3_ | P 6_1_ 2 2 |
| Unit cell (Å) | 55.77 55.77 195.82 | 69.30 69.30 146.06  γ = 60 |
| Total reflections | 197211 (16249) | 614896 (41704) |
| Unique reflections | 14855 (1423) | 17029 (1582) |
| Multiplicity | 13.3 (11.4) | 36.1 (26.4) |
| Completeness (%) | 99.9 (100) | 99.5 (95.0) |
| Mean I/sigma(I) | 25.6 (6.3) | 45.7 (5.4) |
| Wilson B-factor (Å^2^) | 37.75 | 30.50 |
| R-merge | 0.068 (0.389) | 0.06477 (0.7771) |
| R-meas | 0.070 (0.397) | 0.06572 (0.7923) |
| R-pim | 0.019 (0.115) | 0.01096 (0.1508) |
| Reflections used in refinement | 17028 (1582) | 17028 (1582) |
| Reflections used for R-free | 852 (79) | 852 (79) |
| R-work | 0.2150 (0.2720) | 0.2208 (0.2656) |
| R-free | 0.2731 (0.3242) | 0.2447 (0.3060) |
| Number of non-hydrogen atoms | 4848 | 1308 |
| macromolecules | 4684 | 1217 |
| solvent | 164 | 91 |
| Protein residues | 572 | 149 |
| RMS (bonds (Å)) | 0.012 | 0.012 |
| RMS (angles (^o^)) | 1.317 | 1.450 |
| Ramachandran favored (%) | 95.68 | 95.24 |
| Ramachandran allowed (%) | 4.32 | 4.76 |
| Ramachandran outliers (%) | 0.0 | 0.0 |
| Average B-factor (Å^2^) | 49.53 | 34.82 |
| Macromolecules | 49.52 | 34.21 |
| Solvent | 50.77 | 42.99 |

*Statistics for the highest-resolution shell are shown in parentheses
